# Efficient Maturation and Cytokine Production of Neonatal DCs Requires Combined Proinflammatory Signals

**DOI:** 10.1080/17402520500116772

**Published:** 2005-06

**Authors:** Doreen Krumbiegel, Jan Rohr, Peter Schmidtke, Markus Knuf, Fred Zepp, Claudius U. Meyer

**Affiliations:** Department of Pediatric Immunology and Infectious Diseases Children's Hospital Johannes Gutenberg University Mainz 55131 Germany

## Abstract

Specific functional properties of dendritic cells (DCs) have been suspected as being responsible for the impaired specific immune responses observed in human neonates. To analyze stimulatory requirements for the critical transition from immature, antigen-processing DCs to mature, antigen-presenting DCs, we investigated the effect of different proinflammatory mediators and antigens on phenotype and cytokine secretion of human neonatal DCs derived from hematopoietic progenitor cells (HPCs). Whereas single proinflammatory mediators were unable to induce the maturation of neonatal DCs, various combinations of IFNγ, CD40L, TNFα, LPS and antigens, induced the maturation of neonatal DCs documented by up-regulation of HLA-DR, CD83 and CD86. Combinations of proinflammatory mediators also increased cytokine secretion by neonatal DCs. Especially combined stimulation with LPS and IFNγ proved to be very efficient in inducing maturation and cytokine synthesis of neonatal DCs. In conclusion, neonatal DCs can be stimulated to express maturation as well as costimulatory surface molecules. However, induction of maturation requires combined stimulation with multiple proinflammatory signals.


*Clinical & Developmental Immunology*, June 2005; 12(2): 99–105

Taylor & Francis

Taylor & Francis Group

### Efficient maturation and cytokine production of neonatal DCs requires combined proinflammatory signals

DOREEN KRUMBIEGEL, JAN ROHR, PETER SCHMIDTKE, MARKUS KNUF, FRED ZEPP, & CLAUDIUS U. MEYER

*Department of Pediatric Immunology and Infectious Diseases, Children's Hospital, Johannes Gutenberg University, 55131 Mainz, Germany*

**Abstract**

Specific functional properties of dendritic cells (DCs) have been suspected as being responsible for the impaired specific immune responses observed in human neonates. To analyze stimulatory requirements for the critical transition from immature, antigen-processing DCs to mature, antigen-presenting DCs, we investigated the effect of different proinflammatory mediators and antigens on phenotype and cytokine secretion of human neonatal DCs derived from hematopoietic progenitor cells (HPCs). Whereas single proinflammatory mediators were unable to induce the maturation of neonatal DCs, various combinations of IFNγ, CD40L, TNFα, LPS and antigens, induced the maturation of neonatal DCs documented by up-regulation of HLA-DR, CD83 and CD86. Combinations of proinflammatory mediators also increased cytokine secretion by neonatal DCs. Especially combined stimulation with LPS and IFNγ proved to be very efficient in inducing maturation and cytokine synthesis of neonatal DCs. In conclusion, neonatal DCs can be stimulated to express maturation as well as costimulatory surface molecules. However, induction of maturation requires combined stimulation with multiple proinflammatory signals.

**Keywords:** *Cytokines, dendritic cells (DCs), hematopoietic progenitor cells (HPCs), human, inflammation, maturation*

### Introduction

Clinical experience shows that early infancy is associated with an increased susceptibility to infectious diseases as well as impaired responses to vaccines. These findings have been attributed to the immaturity of the neonatal immune system. The developmental status of the specific immune system in human newborns entails significant differences to the immunological capacity seen in adolescents or adults. While some characteristics of neonatal T cells contribute to the immunological immaturity (Adkins et al. 2001), it has been proposed that an impaired function of antigen-presenting cells might also play an important role (Trivedi et al. 1997). Data from murine models showed an impaired ability of neonatal DCs to activate naïve T lymphocytes in comparison to DCs from adult animals (Muthukkumar et al. 2000). In contrast, conflicting data has been published concerning the functional capacity of human neonatal DCs. While some authors report that DCs generated from human newborns are potent stimulators of allogenous T cells (Sorg et al. 1998), others observe an impaired function of neonatal DCs compared to adult DCs (Hunt et al. 1994). Only the inability of neonatal DCs to deliver the signals needed for the polarization of immune responses towards a $T_H$1-type has been reported consistently (Taylor and Bryson 1985, Trivedi et al. 1997, Delespesse et al. 1998).

We investigated the effect of different proinflammatory mediators as well as viral and bacterial antigens on maturation and secretory function of neonatal hematopoietic progenitor cell (HPC)-derived DCs. The maturational status of DCs was determined by the expression pattern of surface molecules and the production of cytokines.

Correspondence: D. Krumbiegel, Pediatric Immunology, Children's Department, University Hospital Mainz, Obere Zahlbacher Str. 63, 55131 Mainz, Germany. Tel: 49 6131 393 3331. Fax: 49 6131 393 3424. E-mail: krumbieg@uni-mainz.de

ISSN 1740-2522 print/ISSN 1740-2530 online © 2005 Taylor & Francis Group Ltd
DOI: 10.1080/17402520500116772

While reports suggest that neonatal and adult APCs synthesize equivalent amounts of IL-12p40 in response to microbial stimuli, secretion of IL-12p70 by neonatal APCs is generally thought to be defective (Lee et al. 1996, Joyner et al. 2000, Goriely et al. 2001, Tonon et al. 2002, Langrish et al. 2002). Since IL-12 plays a crucial role for the polarization of immune responses towards a $T_H1$-type, we studied the production of both the IL-12p40 sub-unit and the bioactive IL-12p70 heterodimer by neonatal DCs after stimulation with proinflammatory mediators.

### Materials and methods

### *Isolation and culture of cord blood CD34$^+$ HPCs*

Umbilical cord blood samples were collected according to institutional guidelines by venipuncture of placental blood from healthy full-term infants born by cesarean section. The heparinized blood was immediately centrifuged to obtain autologous plasma and then diluted 1:1 with RPMI 1640 (Gibco, Eggenstein, Germany). Plasma samples were heat inactivated at 56°C for 30 min and stored at −80°C. The mononuclear cell fraction (CBMCs) was obtained by Ficoll-Hypaque (Biochrom seromed, Berlin, Germany) density centrifugation. CBMCs were washed and magnetically labeled for CD34 expression with anti-CD34 paramagnetic Microbeads (Miltenyi-Biotec, Bergisch-Gladbach, Germany). Labeled cells were enriched on VS + Separation Columns™ in the magnetic field of a VarioMACS™ (both Miltenyi Biotec, Bergisch-Gladbach, Germany) according to the manufacturer's instructions. For increased purity of enriched CD34$^+$ HPCs, the separation step of each sample was performed twice.

Enriched CD34$^+$ HPCs ($1 \times 10^5$ cells/well) were amplified in culture medium (RPMI 1640; Gibco, Eggenstein, Germany) containing 2 mM L-glutamine (Gibco, Eggenstein, Germany), 1 mM sodium pyruvate, 100 IU/ml penicillin, 100 µg/ml streptomycin, 1 × MEM amino acids (Biochrom, Berlin, Germany), 0,05 mM β-mercaptoethanol (Sigma-Aldrich, Taufkirchen, Germany) with 10% AB-Serum (PAN Biotech, Aidenbach, Germany), 50 ng/ml rhFLT3-Ligand, 40 ng/ml rhSCF and 20 ng/ml rhTPO (Tebu, Offenbach, Germany) in 24-well flat-bottom plates (Nunc, Wiesbaden, Germany). Cells were incubated at 37°C in a humidified atmosphere containing 5% $CO_2$. Medium and cytokines were replaced every 3 days until the start of stimulation assays, which was defined as day 0.

### *Generation of dendritic cells (DCs) from HPCs*

Differentiation into immature DCs was induced by culturing amplified HPCs ($5 \times 10^5$ cells/well) with culture medium containing 10% autologous plasma, 100 ng/ml rhGM-CSF and 20 ng/ml rhIL-4 in 24-well flat-bottom plates (Nunc, Wiesbaden, Germany) for 48 h. Subsequently, fresh medium and proinflammatory stimulators were added at day 2 by carefully replacing half of the supernatants for the following 72 h, including rhIFNγ (50 ng/ml), rhTNFα (20 ng/ml), human soluble CD40L (500 ng/ml; all from Tebu, Offenbach, Germany), LPS from *E. coli* (10 µg/ml; Sigma-Aldrich, Taufkirchen, Germany) and/or a mixture containing inactivated complete viruses (*Measles Virus*, *Edmonston* 1 µg/ml; *Respiratory Syncytial Virus* (Long) 20 µg/ml and *Parainfluenza 3* (C234) 10 µg/ml (all from Chemicon, Hofheim, Germany).

### *Flow cytometric immunophenotyping*

DC populations were characterized by labeling with fluorochrome-conjugated monoclonal Antibodies (mAbs) and analyzed by flow cytometry (FACSCalibur™, CellQuest™, Becton Dickinson, Heidelberg, Germany). Antibodies used were: IgG1 and IgG2a isotype control mAbs, HLA-DR (all from Becton Dickinson, Heidelberg, Germany), CD11c (Abcam, Cambridge, UK), CD83 and CD86 (Coulter Imunotech, Hamburg, Germany).

### *Detection of cytokines in culture supernatants*

Cell culture supernatants were collected at several points in time from amplified HPCs (day 0), from immature DCs after a 48 h incubation period with GM-CSF and IL-4 (day 2) and after an additional 72 h incubation period with GM-CSF, IL-4 with or without different proinflammatory cytokines and/or antigen (day 5). Supernatants were stored at −80°C until use. IL-12p40 concentration was measured by enzyme-linked immunosorbent assay (ELISA) according to the manufacturer's instructions (OptEIA™ ELISA Set, Becton Dickinson, Heidelberg, Germany). The concentrations of IL-1β, IL-6, IL-8, IL-10, IL-12p70 and TNFα were quantified with the Human Inflammation Cytometric Bead Array Kit (CBA, Becton Dickinson, Heidelberg, Germany) according to the manufacturer's instructions.

### Results

### *Maturation of neonatal DCs*

After the amplification period (day 0), HPCs showed a low expression level of the myeloid DC marker CD11c and the costimulatory molecule CD86. Furthermore, a moderate expression of the MHC class II HLA-DR and the absence of the DC maturation marker CD83, were found (Figure 1).

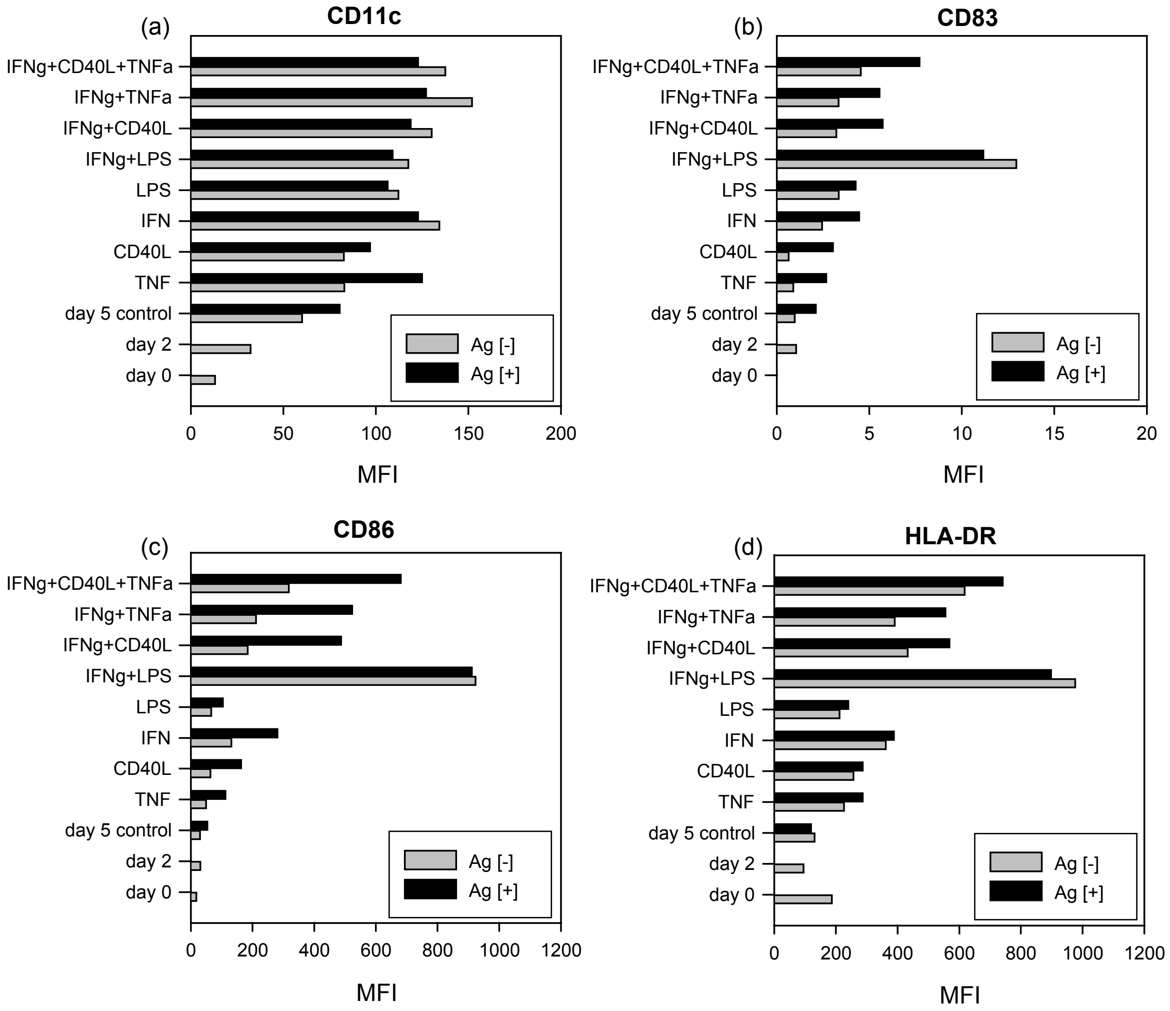

Figure 1. Flow cytometric analysis of CD11c (a), CD83 (b), CD86 (c), HLA-DR (d). Amplified HPCs (day 0) were cultured for 2 days in culture medium containing 10% autologous plasma, GM-CSF and IL-4 (day 2). Cells were then cultured for another 72 h with GM-CSF/IL-4 and different proinflammation cytokine combinations and with or without viral antigens (Measles, RSV, Parainfluenza)—(day 5). This is the mean of five experiments.

Incubation of the HPCs with GM-CSF and IL-4 for 48 h resulted in a moderately up-regulated expression of CD11c, whereas the expression of CD86, HLA-DR and CD83 remained very low (day 2). This phenotype is consistent with the reported state of immature DCs.

In addition to GM-CSF and IL-4, immature neonatal DCs were supplemented with proinflammatory mediators and/or viral antigens at day 2 for a further 72 h in order to induce the maturation (day 5). Exposure to GM-CSF and IL-4 alone, as well as exposure to each of the proinflammatory mediators and/or antigens, resulted in increased CD11c expression of cultured cells at day 5 (Figure 1a). Whereas the addition of single proinflammatory mediators only led to small increases in the expression of CD83, CD86 and HLA-DR, combined stimuli induced a strong increase in marker expression at day 5 (Figure 1b–d). Especially the combination of IFNγ and LPS induced very high expression rates of CD83, CD86 and HLA-DR in neonatal DCs, while neither LPS nor IFNγ alone were able to induce this effect. This synergistic phenomenon was specific for the combination of IFNγ and LPS. Combinations of IFNγ with other mediators such as CD40L or TNFα were less potent inducers of DC maturation. Combination of LPS and IFNγ with other proinflammatory cytokines did not further increase the expression levels of CD83, CD86 and HLA-DR beyond the levels observed after stimulation with LPS and IFNγ (data not shown). In the presence of both antigens and proinflammatory mediators, the expression rate of CD83, CD86 and HLA-DR exceeded those observed with proinflammatory mediators alone.

### *Cytokine secretion of neonatal DCs*

Amplified HPCs (day 0) and immature DCs (day 2) secreted only low amounts of IL-8, IL-12p40 and

TNFα, whereas no IL-1β, IL-6 and IL-12p70 were detectable (Figure 2).

After prolonged incubation with GM-CSF and IL-4 for 72 h, production of IL-6 and IL-8 marginally increased, whereas the secreted amounts of IL-12p40 decreased (day 5). All other cytokine concentrations remained virtually unchanged.

Exposure of immature neonatal DCs to either IFNγ or LPS for 72 h only induced small increments in the synthesis of IL-1β and TNFα in comparison to day 5 controls, whereas the amounts of IL-6 and IL-8 increased considerably (Figure 2). However, combined stimulation with LPS and IFNγ led to a marked increase in secretion of IL-1β, IL-6, IL-8, TNFα, IL-12p40 and IL-12p70 after 72 h. Particularly noteworthy is the production of IL-12p70 after combined stimulation of neonatal DCs with LPS and IFNγ, since it could not be induced with any other mediator alone or mediator combination.

DCs stimulated to mature with IFNγ or combinations of IFNγ with CD40L and/or TNFα, revealed no increase in IL-12p40 expression in comparison to day 5 controls. However, the addition of viral antigens together with IFNγ or combinations with CD40L and/or TNFα, more than doubled IL-12p40 concentration (Figure 2), but did not stimulate IL-12p70 expression. Furthermore, the secretion of IL-1β and TNFα were not increased in the presence of antigens, independent of added stimulators (IFNγ, CD40L, TNFα or combinations of these).

Interestingly, the presence of antigens, LPS or the combination of LPS and IFNγ, induced considerable expression of IL12p40, mostly higher than that seen with IFNγ and combinations. Combination of LPS with other proinflammatory mediators showed no additional increase in cytokine synthesis (data not shown).

In contrast, stimulation with antigens in addition to IFNγ alone or combinations of IFNγ with CD40L and/or TNFα, initiated high cytokine production by neonatal DCs (Figure 2). However, when IFNγ and LPS were present in cultures the addition of antigen decreased the levels of all cytokines examined.

Very high concentrations of IL-8 were detected after stimulation of neonatal DCs with all proinflammatory mediators or their combinations tested. In contrast, neither amplified HPCs, immature DCs nor DCs stimulated with several proinflammatory mediators, expressed IL-10 (data not shown).

### Discussion

Increased susceptibility to infectious diseases in the postnatal phase and in early infancy is a well known clinical phenomenon. In this study, we investigated whether the immaturity of neonatal immune responses might be based on an impairment in the maturation and secretory capacity of DCs by stimulating neonatal immature DCs with various combinations of proinflammatory mediators and antigens.

Since the frequency of DCs is very low in peripheral blood, neonatal DCs were generated *in vitro* from cord blood $CD34^+$ HPCs. Earlier reports describe the successful generation of DCs from HPCs in a FCS-supplemented medium (Santiago-Schwarz et al. 1992, Reid et al. 1992, Arrighi et al. 1999, Cella et al. 1999, Manome et al. 1999, Gagliardi et al. 2000). However, this approach involves the risk of an accidental exposure to foreign antigens and/or mediators and could therefore facilitate the activation of DCs. DCs grown in a medium containing FCS show an increased secretion of IL-12 in comparison to those generated in a medium supplemented with autologous plasma (Ebner et al. 2001). To minimize these unspecific side effects, we modified the protocol described by Arrighi et al. (1999) for the generation of HPC-derived DCs. Instead of FCS we used human AB-serum and autologous plasma, respectively, during the amplification period and during differentiation into DCs. The use of both AB-serum and autologous plasma was necessary because the cord blood samples did not contain sufficient amount of autologous plasma for the whole culture period.

The induction of protective cell-mediated immunity requires an efficient maturation of DCs. *In vivo*, immature DCs at the site of infection are exposed to many different proinflammatory mediators, which may influence the maturation of DCs. Therefore a main target of this study was to determine the effect of different proinflammatory mediators and microbial antigens on the maturation of neonatal DCs *in vitro*. A combination of antigens from three viral pathogens (RSV, PIV, MV) were selected according to their outstanding importance during infancy.

Even though each of the proinflammatory stimuli used in this study have previously been described as efficient signals for the maturation of adult DCs (Santiago-Schwarz et al. 1992, Arrighi et al. 1999, Vieira et al. 2000, Kalinski et al. 2001, de Jong et al. 2002), our results show that exposure to a single proinflammatory mediator is a poor stimulus to induce maturation of neonatal DCs. These observations point towards a lower responsiveness of neonatal DCs to single proinflammatory mediators in comparison to adult DCs.

Cytokine production by DCs plays an important role in the course of immune responses (de Saint-Vis et al. 1998). In our experiments all mediators induced the production of IL-6 und IL-8 in neonatal DCs. The addition of LPS with or without IFNγ supplementary, increased IL-6 synthesis in comparison to other mediators or their combinations. TNFα, IL-6 and IL-8 are known to play important roles in inflammatory processes and also support specific immune responses. IL-6 is a potent growth and

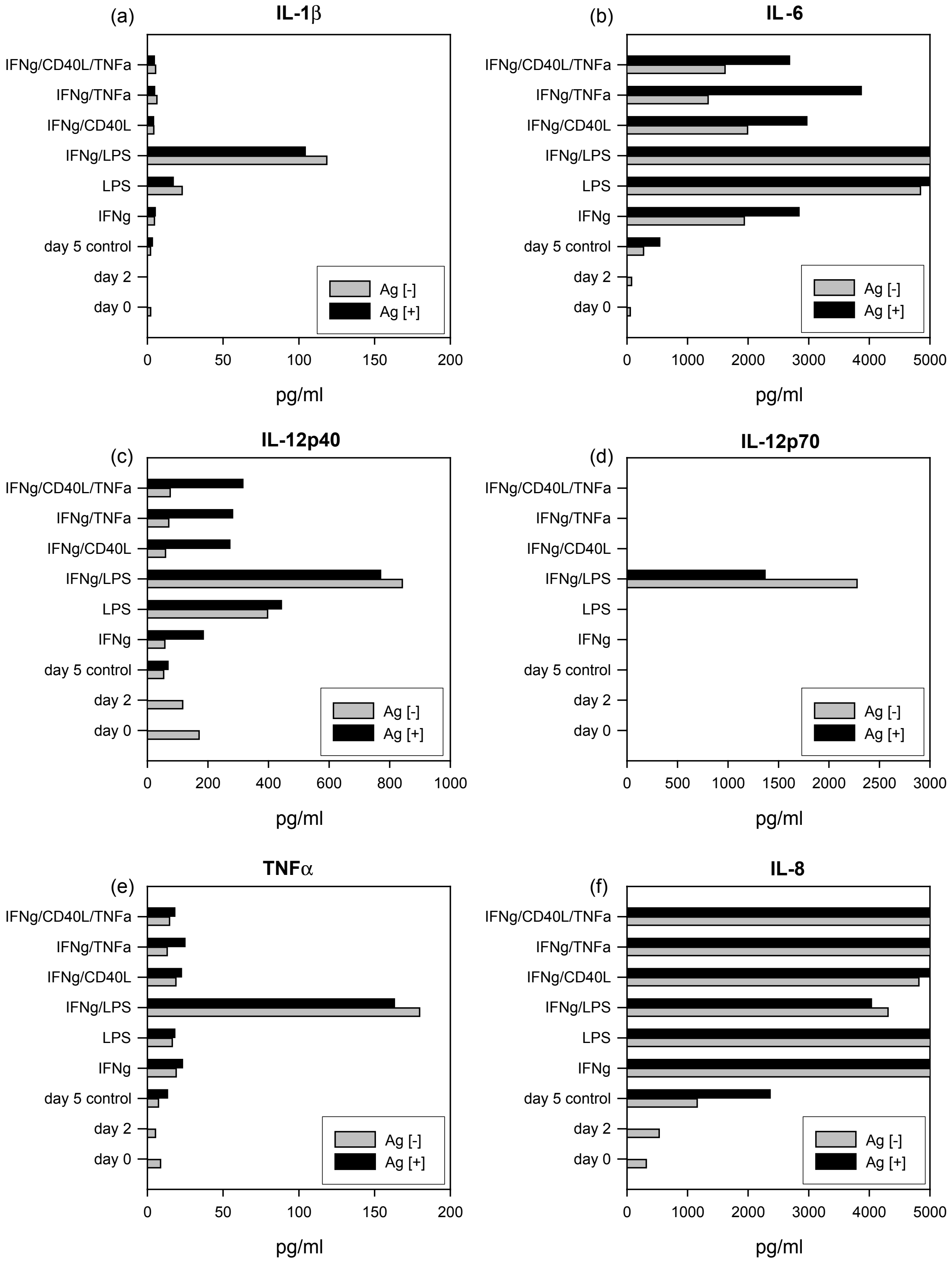

Figure 2. Cytokine expression measured by IL-12p40 ELISA and CBA. Cells were cultured as described before, supernatants were taken after HPC amplification (day 0), from immature DCs (day 2) and from mature DCs (day 5) and frozen at −80°C. This is the mean of four experiments.

differentiation factor for B cells that participates in the development of CTL responses and enhances the survival of naïve T cells (Verhasselt et al. 1997, Teague et al. 2000). IL-8 supports the survival of hematopoietic cells (Han et al. 1997). TNFα production has been described in many types of DCs (Larrick et al. 1989, Caux et al. 1994, Verhasselt et al. 1997). It enhances the survival of hematopoietic stem cells and supports their differentiation into DCs (Morrison et al. 2003). This explains the large number of DCs under inflammatory conditions, where local concentrations of TNFα are high. In our experiments we showed that HPC-derived neonatal DCs are also able to produce TNFα, but only after combined stimulation with LPS and IFNγ.

Cell-mediated immune responses to intracellular pathogens are promoted by Th1 effector cells, which are characterized by their production of IFNγ. However, IFNγ itself is not sufficient for the development of a Th1 immune response. The dominant factor for the development of Th1 immune responses is thought to be the secretion of IL-12 by DCs (Trinchieri 1994). While reports suggest that neonatal and adult PBMCs synthesize equivalent amounts of IL-12p40 in response to microbial stimuli (Scott et al. 1997), the secretion of bioactive IL-12p70 by cord blood cells is generally demonstrated to be limited (Lee et al. 1996, Joyner et al. 2000, Goriely et al. 2001, Tonon et al. 2002, Langrish et al. 2002). We observed that only DCs matured with LPS with or without IFNγ, but no other proinflammatory mediator or their combinations, profoundly increased IL-12p40 production in comparison to day 5 controls. Whereas antigen addition doubled IL-12p40 secretion, it did not increase but dampened IL-12p70 synthesis, drawing the conclusion that the antigen combination used selectively activates IL-12p40 gene expression. Upon maturation, neonatal DCs have been shown to be characterized by a defect in IL-12p35 gene expression in comparison to adult DCs. This limited production of IL-12p35 has been described as being stimuli-independent (CD40L, LPS, Poly (I:C), IFNγ), which could however, be overcome by the combination of LPS and IFNγ (Goriely et al. 2001). Adult DCs have been described to produce IL-12p70 after stimulation with LPS alone, but not with TNFα or IFNγ (Cella et al. 1999). Interestingly, the addition of IFNγ, with or without TNFα, induced an increase in the expression of maturation and costimulatory surface molecules without induction of IL-12p70 production. This observation suggests that phenotypically mature DCs do not necessarily synthesize IL-12p70.

During the maturation of DCs, a decreased viability after stimulation with several proinflammatory substances was observed by flow cytometry (data not shown). Since proinflammatory mediators and antigens also induced DC maturation, it could be suspected that maturation of DCs increases their susceptibility to apoptosis. A question arising from these observations is whether the maturation of neonatal DCs may be influenced by the exposure to mediators released by apoptotic and/or necrotic cells. However, at least after addition of supernatants of apoptotic and/or necrotic autologous cells to immature DCs, the expression pattern of CD11c, CD86, CD83 and HLA-DR did not show any signs of changes in DC maturation status (data not shown).

In conclusion, the combination of proinflammatory mediators was far more efficient in inducing the maturation and cytokine secretion of neonatal DCs in comparison to single proinflammatory agents. The synergistic effect of combinations of proinflammatory mediators on the maturational state of neonatal DCs could best be exemplified by the use of IFNγ and LPS. The combined stimulation of immature neonatal DCs with IFNγ and LPS efficiently induced high expression rates of maturation and costimulatory markers as well as production of proinflammatory cytokines. These findings indicate a critical importance for combined signaling of microbial and proinflammatory mediators in the maturational process of neonatal DCs. Furthermore, they suggest a high activation threshold of neonatal DCs, which could contribute to the clinical observation of an increased susceptibility to infectious diseases.

### Acknowledgements

This work was supported by the Mainzer Forschungsförderungsprogramm (MaiFor).

### References

Adkins B, Bu Y, Guevara P. 2001. The generation of Th memory in neonates versus adults: Prolonged primary Th2 effector function and impaired development of Th1 memory effector function in murine neonates. J Immunol 166:918–925.

Arrighi JF, Hauser C, Chapuis B, Zubler RH, Kindler V. 1999. Long-term culture of human CD34(+) progenitors with FLT3-ligand, thrombopoietin, and stem cell factor induces extensive amplification of a CD34(−)CD14(−) and a CD34(−)CD14(+) dendritic cell precursor. Blood 93: 2244–2252.

Caux C, Vanbervliet B, Massacrier C, Azuma M, Okumura K, Lanier LL, Banchereau J. 1994. B70/B7-2 is identical to CD86 and is the major functional ligand for CD28 expressed on human dendritic cells. J Exp Med 180:1841–1847.

Cella M, Salio M, Sakakibara Y, Langen H, Julkunen I, Lanzavecchia A. 1999. Maturation, activation, and protection of dendritic cells induced by double-stranded RNA. J Exp Med 189:821–829.

Delespesse G, Yang LP, Ohshima Y, et al. 1998. Maturation of human neonatal CD4+ and CD8+ T lymphocytes into Th1/Th2 effectors. Vaccine 16:1415–1419.

Ebner S, Ratzinger G, Krosbacher B, et al. 2001. Production of IL-12 by human monocyte-derived dendritic cells is optimal when the stimulus is given at the onset of maturation, and is further enhanced by IL-4. J Immunol 166:633–641.

Gagliardi MC, Sallusto F, Marinaro M, Langenkamp A, Lanzavecchia A, De Magistris MT. 2000. Cholera toxin induces maturation of human dendritic cells and licences them for Th2 priming. Eur J Immunol 30:2394–2403.

Goriely S, Vincart B, Stordeur P, et al. 2001. Deficient IL-12(p35) gene expression by dendritic cells derived from neonatal monocytes. J Immunol 166:2141–2146.

Han ZC, Lu M, Li J, et al. 1997. Platelet factor 4 and other CXC chemokines support the survival of normal hematopoietic cells and reduce the chemosensitivity of cells to cytotoxic agents. Blood 89:2328–2335.

Hunt DW, Huppertz HI, Jiang HJ, Petty RE. 1994. Studies of human cord blood dendritic cells: Evidence for functional immaturity. Blood 84:4333–4343.

de Jong EC, Vieira PL, Kalinski P, et al. 2002. Microbial compounds selectively induce Th1 cell-promoting or Th2 cell-promoting dendritic cells *in vitro* with diverse th cell-polarizing signals. J Immunol 168:1704–1709.

Joyner JL, Augustine NH, Taylor KA, La Pine TR, Hill HR. 2000. Effects of group B streptococci on cord and adult mononuclear cell interleukin-12 and interferon-gamma mRNA accumulation and protein secretion. J Infect Dis 182:974–977.

Kalinski P, Vieira PL, Schuitemaker JH, de Jong EC, Kapsenberg ML. 2001. Prostaglandin E(2) is a selective inducer of interleukin-12 p40 (IL-12p40) production and an inhibitor of bioactive IL-12p70 heterodimer. Blood 97: 3466–3469.

Langrish CL, Buddle JC, Thrasher AJ, Goldblatt D. 2002. Neonatal dendritic cells are intrinsically biased against Th-1 immune responses. Clin Exp Immunol 128:118–123.

Larrick JW, Morhenn V, Chiang YL, Shi T. 1989. Activated Langerhans cells release tumor necrosis factor. J Leukoc Biol 45:429–433.

Lee SM, Suen Y, Chang L, et al. 1996. Decreased interleukin-12 (IL-12) from activated cord versus adult peripheral blood mononuclear cells and upregulation of interferon-gamma, natural killer, and lymphokine-activated killer activity by IL-12 in cord blood mononuclear cells. Blood 88:945–954.

Manome H, Aiba S, Tagami H. 1999. Simple chemicals can induce maturation and apoptosis of dendritic cells. Immunology 98:481–490.

Morrison 3rd, RS, Cruse JM, Wang H, Lewis RE. 2003. Dendritic cell differentiation and proliferation: Enhancement by tumor necrosis factor-alpha. Exp Mol Pathol 75:228–237.

Muthukkumar S, Goldstein J, Stein KE. 2000. The ability of B cells and dendritic cells to present antigen increases during ontogeny. J Immunol 165:4803–4813.

Reid CD, Stackpoole A, Meager A, Tikerpae J. 1992. Interactions of tumor necrosis factor with granulocyte-macrophage colony-stimulating factor and other cytokines in the regulation of dendritic cell growth *in vitro* from early bipotent CD34+ progenitors in human bone marrow. J Immunol 149:2681–2688.

de Saint-Vis B, Fugier-Vivier I, Massacrier C, et al. 1998. The cytokine profile expressed by human dendritic cells is dependent on cell subtype and mode of activation. J Immunol 160:1666–1676.

Santiago-Schwarz F, Belilos E, Diamond B, Carsons SE. 1992. TNF in combination with GM-CSF enhances the differentiation of neonatal cord blood stem cells into dendritic cells and macrophages. J Leukoc Biol 52:274–281.

Scott ME, Kubin M, Kohl S. 1997. High level interleukin-12 production, but diminished interferon-gamma production, by cord blood mononuclear cells. Pediatr Res 41:547–553.

Sorg RV, Kogler G, Wernet P. 1998. Functional competence of dendritic cells in human umbilical cord blood. Bone Marrow Transplant 22:52–54.

Taylor S, Bryson YJ. 1985. Impaired production of gamma-interferon by newborn cells *in vitro* is due to a functionally immature macrophage. J Immunol 134:1493–1497.

Teague TK, Schaefer BC, Hildeman D, et al. 2000. Activation-induced inhibition of interleukin 6-mediated T cell survival and signal transducer and activator of transcription 1 signaling. J Exp Med 191:915–926.

Tonon S, Goriely S, Aksoy E, et al. 2002. Bordetella pertussis toxin induces the release of inflammatory cytokines and dendritic cell activation in whole blood: Impaired responses in human newborns. Eur J Immunol 32:3118–3125.

Trinchieri G. 1994. Interleukin-12: A cytokine produced by antigen-presenting cells with immunoregulatory functions in the generation of T-helper cells type 1 and cytotoxic lymphocytes. Blood 84:4008–4027.

Trivedi HN, HayGlass KT, Gangur V, Allardice JG, Embree JE, Plummer FA. 1997. Analysis of neonatal T cell and antigen presenting cell functions. Hum Immunol 57:69–79.

Verhasselt V, Buelens C, Willems F, De Groote D, Haeffner-Cavaillon N, Goldman M. 1997. Bacterial lipopolysaccharide stimulates the production of cytokines and the expression of costimulatory molecules by human peripheral blood dendritic cells: Evidence for a soluble CD14-dependent pathway. J Immunol 158:2919–2925.

Vieira PL, de Jong EC, Wierenga EA, Kapsenberg ML, Kalinski P. 2000. Development of Th1-inducing capacity in myeloid dendritic cells requires environmental instruction. J Immunol 164:4507–4512.

